# Vital roles of m^5^C RNA modification in cancer and immune cell biology

**DOI:** 10.3389/fimmu.2023.1207371

**Published:** 2023-05-31

**Authors:** Xinyu Gu, Xiao Ma, Chao Chen, Jun Guan, Jing Wang, Shanshan Wu, Haihong Zhu

**Affiliations:** ^1^ State Key Laboratory for Diagnosis and Treatment of Infectious Diseases, National Clinical Research Center for Infectious Diseases, National Medical Center for Infectious Diseases, Collaborative Innovation Center for Diagnosis and Treatment of Infectious Diseases, The First Affiliated Hospital, Zhejiang University School of Medicine, Hangzhou, China; ^2^ Zhejiang University School of Medicine, Hangzhou, Zhejiang, China

**Keywords:** RNA modification, m^5^C, cancer, immune cells, cancer immunity

## Abstract

RNA modification plays an important role in epigenetics at the posttranscriptional level, and 5-methylcytosine (m^5^C) has attracted increasing attention in recent years due to the improvement in RNA m^5^C site detection methods. By influencing transcription, transportation and translation, m^5^C modification of mRNA, tRNA, rRNA, lncRNA and other RNAs has been proven to affect gene expression and metabolism and is associated with a wide range of diseases, including malignant cancers. RNA m^5^C modifications also substantially impact the tumor microenvironment (TME) by targeting different groups of immune cells, including B cells, T cells, macrophages, granulocytes, NK cells, dendritic cells and mast cells. Alterations in immune cell expression, infiltration and activation are highly linked to tumor malignancy and patient prognosis. This review provides a novel and holistic examination of m^5^C-mediated cancer development by examining the exact mechanisms underlying the oncogenicity of m^5^C RNA modification and summarizing the biological effects of m^5^C RNA modification on tumor cells as well as immune cells. Understanding methylation-related tumorigenesis can provide useful insights for the diagnosis as well as the treatment of cancer.

## Introduction

Modifications of biological macromolecules, such as DNA, RNA and proteins, are essential for life. RNA modification at the posttranscriptional level, which does not alter the genome, plays a large role in epigenetics. The first RNA modification site, pseudouridine (Ψ), was discovered in the 1950s (1), and a total of 334 types of RNA modifications have been identified since then (2). Commonly recognized RNA modification sites include N^6^-methyladenosine (m^6^A), 5-methylcytosine (m^5^C), 7-methylguanosine (m^7^G), N^1^-methyladenosine (m^1^A), N^4^-acetylcytidine (ac^4^C), N^6^-acetyladenosine (ac^6^A), pseudouridine (Ψ), uridylation, and phosphorylation (3–5). The deposition, removal and recognition of RNA modification sites are realized through three groups of responsible proteins. “Writers” and “erasers” refer to proteins capable of catalyzing the deposition and removal of a specific RNA modification site, respectively, while “readers”, sometimes also called “binders”, mainly recognize and bind to these modification sites (6, 7). Previous studies have demonstrated that RNA modification occurs not only in messenger RNA (mRNA) but also in noncoding RNAs, such as transfer RNA (tRNA), ribosomal RNA (rRNA), long noncoding RNA (lncRNA), microRNA (miRNA) and small nuclear RNA (snRNA) (8–11). For example, m^6^A and m^5^C modifications of mRNA are crucial in embryo development and stem cell fate determination (12), m^7^G modification of tRNA influences pathogenic infectivity of thermophilic bacteria (13), and m^6^A modification of lncRNA is likely to participate in the process of cell senescence (14).

Among the types of RNA modifications that have been discovered, m^6^A modification is the most widely and comprehensively investigated because of its abundance in eukaryotic cells (15, 16). m^5^C modification, comparatively, is less understood than m^6^A modification, as it is only moderately abundant (2, 17). A study in 2015 discovered that m^5^C consists of approximately 1%, 0.01% and 1% of cytosine residues in the samples extracted from mouse brain, E. coli and HEK293T (human embryonic kidney 293 T) cells (18). However, m^5^C has attracted increasing attention from researchers in recent years as detection methods for m^5^C have progressed (17, 19), showing the presence of m^5^C in mRNA, tRNA, rRNA and viral RNA infecting mammalian cells (20, 21). RNA sequencing methods are commonly used in the detection of m^5^C RNA modification, including RNA bisulphite sequencing, immunoprecipitation-based RNA sequencing and third generation sequencing (21–23). Immunoprecipitation-based RNA sequencing can be further divided into several categories, such as methylated RNA immunoprecipitation sequencing (MeRIP-Seq), 5-azacytidine-mediated RNA immunoprecipitation sequencing (5-azaIP-Seq) and methylation-individual nucleotide resolution crosslinking immunoprecipitation sequencing (miCLIP-Seq) (21, 23). Other detection methods include mass spectrometry, total base composition analysis, nearest neighbor analysis, etc. (24).

Up to now, an increasing amount of evidence has unveiled the importance of m^5^C in the modulation of gene expression, metabolism and diseases (25–27). Specifically, in the field of oncology, posttranscriptional RNA modification has been discovered to play important roles in the development and pathological process of various types of cancers since more than half a century ago (10, 28, 29), which introduced a promising new area of mechanistic exploration and therapeutic innovation. Alterations in m^5^C modifications of both coding RNAs and noncoding RNAs are also highly linked to cell proliferation (30, 31), metabolism (32) and tumor metastasis (33, 34) and appear in various kinds of cancer types, such as hepatocellular carcinoma (35), breast cancer (36) and bladder cancer (32). Moreover, m^5^C has vital impacts on different kinds of immune cells, including B cells, T cells, NK cells, granulocytes and macrophages (37, 38). Obviously, the m^5^C-associated biological changes in immune cells are not to be neglected in the process of cancer development, but there is no comprehensive summary regarding the relationship between m^5^C-associated tumorigenesis and alterations in immune cells.

In this review, we provide a novel and holistic review of m^5^C-mediated cancer development by examining the exact mechanisms underlying the oncogenicity of m^5^C RNA modification and summarize the biological effects of m^5^C RNA modification on tumor cells as well as immune cells.

## The mechanism and basic biological functions of m^5^C RNA modification

**Figure 1:**
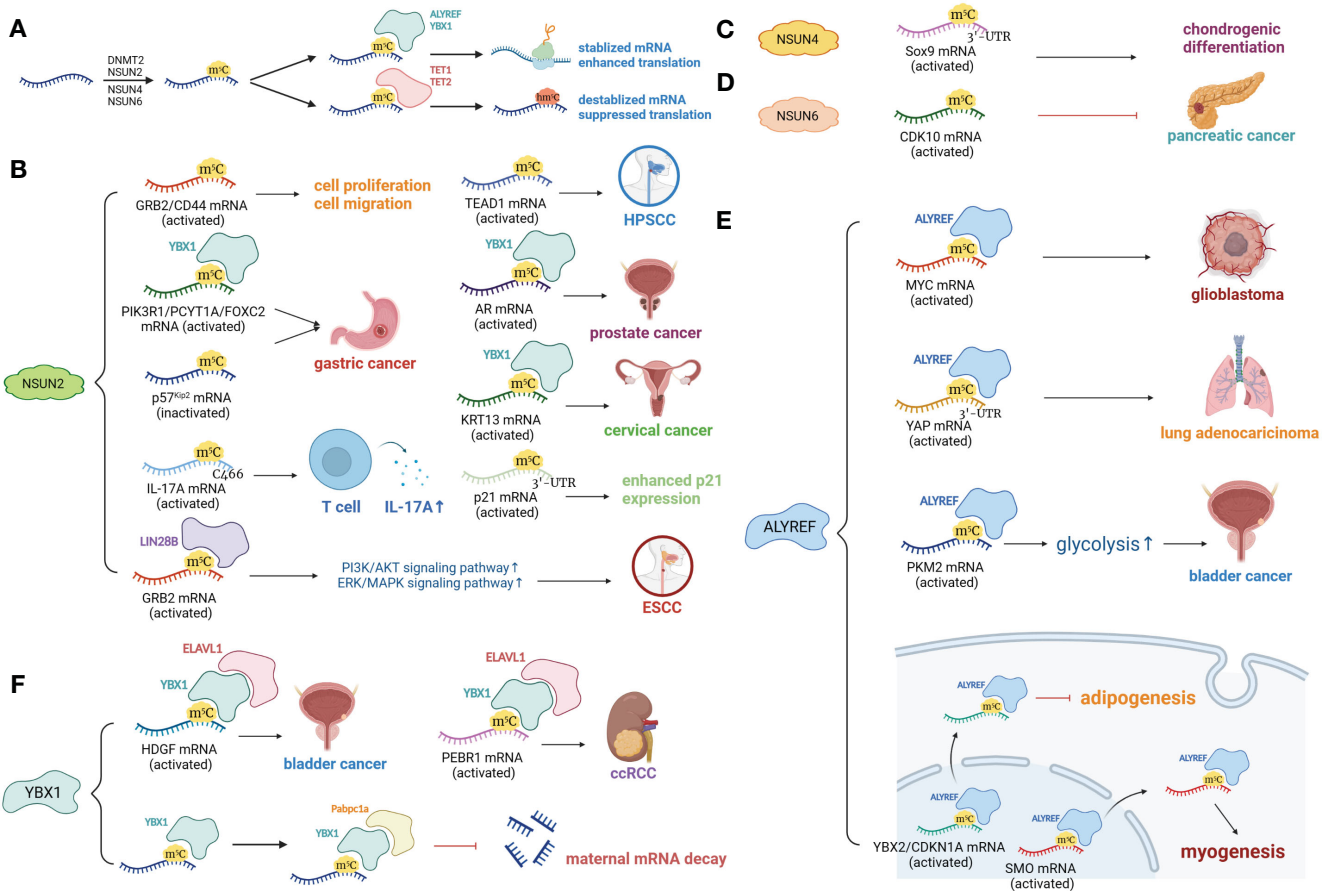
m^5^C modifications of mRNA. **(A)** mRNA is methylated at m^5^C sites by DNMT2, NSUN2, NSUN4 and NSUN6. ALYREF and YBX1 bind and stabilize m^5^C-modulated mRNAs. TET1, TET2 and ALKBH1 remove m^5^C sites by turning them into hm^5^C. **(B)** Target mRNAs of NSUN2 include GRB2, CD44, PIK3R1, PCYTIA, FOXC2, p57Kip2, TEAD1, AR, KRT13, IL-17A, and p21. Most target mRNAs are stabilized due to m^5^C modification, with the exception of p57Kip2, and are associated with enhanced cell proliferation and migration. **(C)** NSUN4-mediated m^5^C at the 3’-UTR of Sox9 mRNA promotes chondrogenic differentiation. **(D)** NSUN6-mediated m^5^C modification suppresses pancreatic cancer by promoting tumor-suppressive CDK10. **(E)** ALYREF binds with MYC, YAP, PKM2 and NEAT1 lncRNA, promoting tumor development and drug resistance. ALYREF facilitates nuclear export of YBX2, CDKN1A and SMO mRNAs, inhibiting adipogenesis and enhancing myogenesis. **(F)** YBX1 binds to m^5^C sites on the FOXC2, HDGF, PEBRQ, AR and KRT13 mRNAs, leading to enhanced mRNA translation and promoting tumorigenesis. YBX1 recognizes and stabilizes m^5^C-modified mRNAs by recruiting Pabpc1a, facilitating maternal-to-zygotic transition.

**Figure 2:**
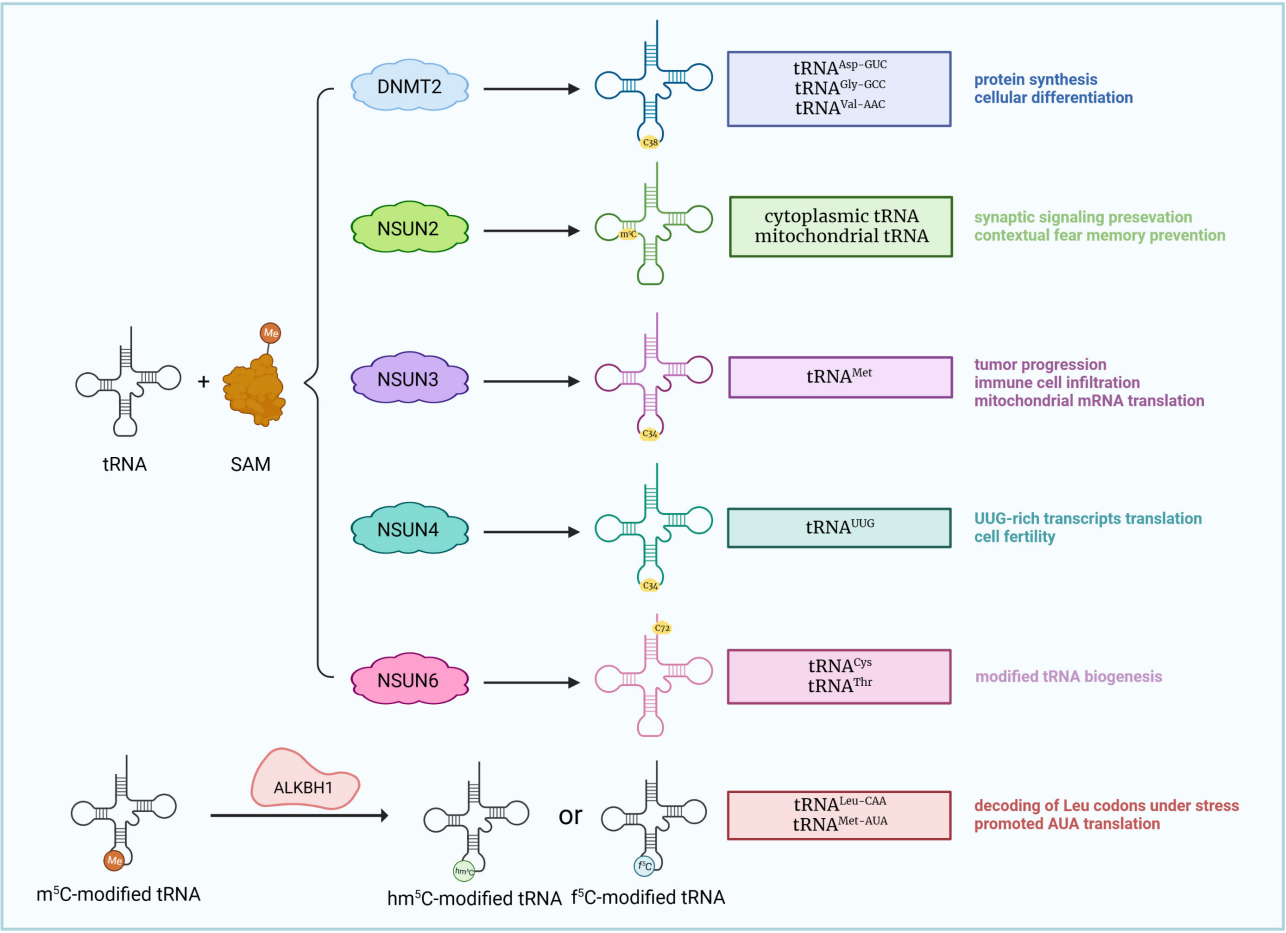
m^5^C modifications of tRNA. SAM induces tRNA m^5^C modification. DNMT2, NSUN2-4, and NSUN6 catalyze m^5^C modification of various tRNAs at different sites, causing distinct biological effects. ALKBH1 removes m5C sites from tRNA^Leu-CAA^ and converts them into f^5^C (5-formylcytidine) sites, promoting the decoding of Leu codons under stress.

**Figure 3:**
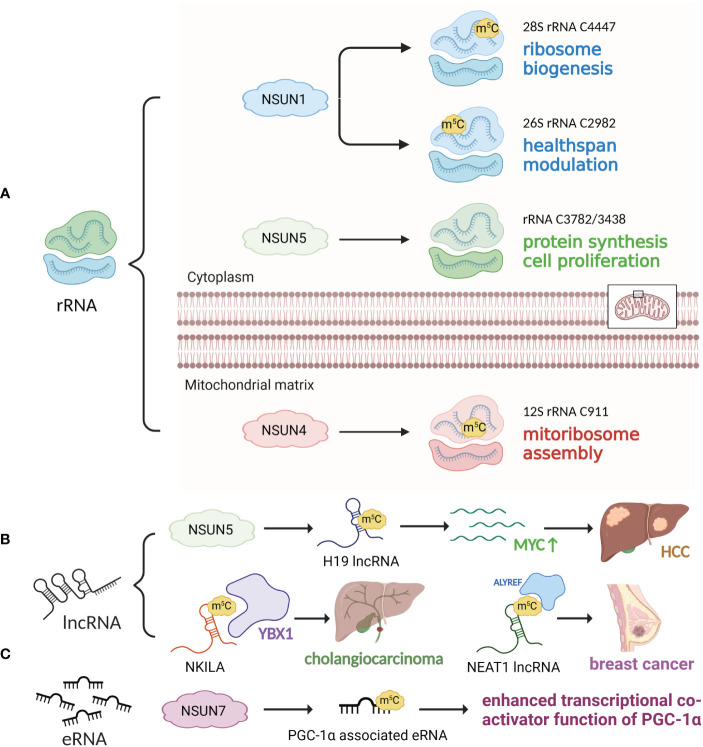
m^5^C modifications of rRNA, lncRNA and eRNA. **(A)** rRNA m^5^C modification is mediated by NSUN1, NSUN4 and NSUN5, facilitating ribosome biogenesis, healthspan modulation, mitoribosomal assembly, protein synthesis and cell proliferation. **(B)** NSUN2 catalyzes m^5^C modification of H19 lncRNA, which stimulates MYC expression and HCC development. YBX1 recognizes and stabilizes m^5^C-modified NKILA, promoting cholangiocarcinoma development. **(C)** NSUN7 m^5^C-methylates eRNA associated with PGC-1α, promoting its transcriptional coactivator function.

There are three main groups of molecular effectors in the process of m^5^C RNA modification, namely, “writers”, “erasers” and “readers” (**Table 1**). “Writers” refer to proteins that facilitate the formation of methylation sites, such as DNMT2 (DNA methyltransferase homolog 2) and the NSUN (NOL1/NOP2/SUN domain) family proteins. “Readers” are related recognition proteins that bind and identify methylation sites, such as ALYREF (Aly/REF export factor) and YBX1(Y-box binding protein 1). Although they do not directly take part in catalysis, the abnormality of “readers” is often associated with metabolic disorders and diseases. “Erasers”, in contrast, facilitate the deletion of methylation sites, such as TET (ten-eleven translocation) family genes and ALKBH1 (AlkB homolog 1), creating a dynamic balance between the two antagonizing biological processes (**Figures 1**–**3**).

**Table 1:** Different types of m^5^C writers, readers and erasers and their biological functions.

Types	Proteins	Target RNAs and m^5^C sites	Cellular functions	Mechanisms	References
Writers	DNMT2	tRNA^Asp-GUC^, tRNA^Gly-GCC^, tRNA^Val-AAC^ (C38 in the anticodon loop)	enhances protein synthesis and cellular differentiation	/	(39–41)
		mRNA	cell proliferation and migration	/	(42)
	NSUN1	28S rRNA (C4447)	ribosome biogenesis	/	(43)
		/		regulates pre-rRNA processing by binding to the 5’-ETS region of pre-rRNA transcript, forming a noncatalytic complex together with box C/D snoRNAs	
		/	cell proliferation	/	(44)
		26S rRNA (C2982)	healthspan modulation	/	(45)
		/	promotes HIV-1 viral latency	competes with HIV-1 Tat protein to interact with HIV-1 TAR RNA	(46)
	NSUN2	tRNA	preserves synaptic signaling at prefrontal cortex pyramidal neurons and suppresses contextual fear memory	/	(47–49)
		mRNA	cell proliferation and migration	m^5^C-methylates GRB2 and CD44	(50)
		mRNA	gastric cancer (GC) development	m^5^C-methylates PIK3R1, PCYT1A and FOXC2 mRNAs; represses p57Kip2 by destabilizing its mRNA in a m^5^C-dependent manner	(30, 34, 51)
		mRNA	esophageal squamous cell carcinoma (ESCC) development	m^5^C-methylates GRB2 *via* LIN28B-dependent way, thus activating PI3K/AKT and ERK/MAPK signaling pathway; promotes TIGAR	(52, 53)
		lncRNA	hepatocellular carcinoma (HCC) development	m^5^C-methylates H19 lncRNA, leading to MYC stimulation; modulates Ras signaling pathway and cell cycle	(35, 54)
		mRNA	hypopharyngeal squamous cell carcinoma (HPSCC) development	m^5^C-methylates TEAD1 mRNA, thus upregulating its expression	(55)
		mRNA	prostate cancer development	m^5^C-methylates and stabilizes androgen receptor (AR) mRNA	(56)
		mRNA	cervical cancer development	m^5^C-methylates KRT13 mRNA, enhancing its binding with m^5^C reader YBX1	(57)
		/	nasopharyngeal carcinoma (NPC) development	negatively regulates immune cell infiltration in tumor microenvironment (TME)	(58)
		/	uveal melanoma development	/	(59)
		mRNA (C466)	enhances IL-17A secretion of T cells	m^5^C-methylates IL-17A mRNA in T cells	(60)
		mRNA	enhances p21 expression under conditions of oxidative stress-induced cellular senescence	m^5^C-methylates p21 mRNA at the 3’-UTR	(61)
		/	promotes ALYREF’s nuclear-cytoplasmic shuttling, RNA-binding affinity and associated mRNA export	/	(62)
	NSUN3	/	possibly promotes low-grade glioma development	/	(63)
		/	promotes the development of head and neck squamous cell carcinoma (HNSCC)	promotes tumor progression by regulating immune cell infiltration	(64)
		tRNA^Met^ (C34 at the anticodon loop)	facilitates mitochondrial mRNA translation, thus promoting metastasis	produces methylated tRNA^Met^ needed for initiation and elongation of mitochondrial mRNA translation	(27, 65)
		/	facilitates CD8+ T cells infiltration	/	(66)
		/	facilitates M2 macrophages infiltration	/	(64)
		/	preserves mitochondrial functions	/	(67)
	NSUN4	12S rRNA (C911)	facilitates mitoribosomal assembly	m^5^C-methylates 12S rRNA and interacts with MTERF4	(68)
		tRNA (C34)	facilitates adaptation to higher temperatures	ensures translation efficiency of UUG-rich transcripts and fertility	(69)
		mRNA	chondrogenic differentiation	m^5^C-methylates the 3’-UTR of Sox9 mRNA	(70)
		/	HCC development	/	(71, 72)
		/	neutrophil infiltration	/	(66)
	NSUN5	rRNA (C3782 in human and C3438 in mice)	protein synthesis and cell proliferation	/	(73, 74)
		/	HCC development	strengthens ribosome functions and global protein translation	(75)
		/	colorectal cancer (CRC) development	/	(76)
	NSUN6	tRNA^Cys^, tRNA^Thr^ (C72)	/	/	(77, 78)
		/	suppresses triple-negative breast cancer (TNBC)	potential regulation of infiltration of CD4+ T cells	(36)
		mRNA	suppresses pancreatic cancer	promotes tumor-suppressive CDK10	(31)
		mRNA	suppresses testis, thyroid and ovary cancers	higher expression and translation levels of m^5^C-methylated mRNAs	(79)
		/	CRC development	/	(80, 81)
		mRNA	promotes cell cycle dysfunction	/	(82)
		/	infiltration of B cells and CD8+ T cells	/	(80)
		mRNA	formation of antibody-secreting plasma cells	/	(83)
	NSUN7	eRNA	enhances transcriptional coactivator function of PGC-1α	m^5^C-methylates eRNA associated with PGC-1α	(84)
Readers	ALYREF	/	HCC development	promotes eIF4A3 expression; disrupts cell cycle and mitosis regulation	(72, 85, 86)
		mRNA	glioblastoma development	stabilizes MYC mRNA; activates the Wnt/β-catenin signaling pathway	(87, 88)
		/	glioma development	/	(63)
		/	neuroblastoma development	forms a nuclear coactivator complex with MYCN to stimulate USP3 transcription	(89)
		mRNA (3’-UTR)	lung adenocarcinoma development	binds with 3’-UTR of YAP mRNA, increasing its stability and thus enhancing exosome secretion, tumor malignancy and drug resistance	(90, 91)
		/	HNSCC development	enhances mitochondrial activity and intracellular energy metabolism, ensuring continuous energy supplies	(92, 93)
		mRNA	bladder cancer development	binds and stabilizes PKM2 mRNA, enhancing PKM2-mediated glycolysis	(32)
		lncRNA	breast cancer development	binds with the NEAT1 lncRNA promoter region, enhancing its transcription	(94, 95)
		/	suppresses colon adenocarcinoma development	/	(81)
		mRNA	inhibits adipogenesis	recognizes and exports YBX2 and CDKN1A mRNAs into the cytoplasm, leading to increased YBX2 and CDKNIA protein expression levels which inhibit adipogenesis	(96, 97)
		mRNA	promotes myogenesis	recognizes and exports SMO mRNA into the cytoplasm, leading to increased SMO protein expression levels which promote myogenesis	(97)
		/	promotes retrovirus replication	/	(98)
		/	possibly promotes abdominal aortic aneurysm (AAA) and infiltration of CD45+ leukocytes and CD3+ T cells	/	(99)
	YBX1	mRNA	GC development	recognizes and binds with NSUN2-mediated m^5^C sites on FOXC2 mRNA to stabilize it	(51)
		mRNA	bladder cancer development	stabilizes oncogenic HDGF mRNA by targeting the m^5^C-modified site on its 3’-UTR and recruiting ELAVL1	(100)
		/	glioblastoma development	/	(101)
		/	CRC development	/	(76, 102)
		lncRNA	cholangiocarcinoma development	recognizes and stabilizes m^5^C-modified NKILA	(103)
		mRNA	suppresses the development of ccRCC	YBX1/ELAVL1 complex binds and stabilizes PEBR1 mRNA, which negatively modulates ccRCC	(104)
		mRNA	prostate cancer development	recognizes and binds with NSUN2-mediated m^5^C sites on AR mRNA to stabilize it	(56)
		/	epithelial ovarian cancer development	modulates the expression of a variety of downstream targets, including CD44, thus enhancing chemoresistance	(105)
		mRNA	cervical cancer development	recognizes and binds with NSUN2-mediated m^5^C sites on KRT13 mRNA to stabilize it	(57)
		/	embryonic brain development	/	(101)
		mRNA	facilitates the maternal-to-zygotic transition	recognizes and stabilizes m^5^C-modified mRNAs by recruiting Pabpc1a, preventing maternal mRNA decay	(106)
Erasers	TET1	mRNA	ensures proper completion of DNA repair and survival of cells after DNA damage	mediates mRNA m^5^C-demethylation, thus promoting mRNA-dependent recombination	(107)
	TET2	/	convert m^5^C into hm^5^C	decreases m^5^C	(108, 109)
		/	possibly promotes low-grade glioma	/	(63)
		/	possibly suppresses the development of ccRCC	/	(110, 111)
		/	possibly inhibits ovarian cancer	/	(112)
		/	possibly inhibits prostate adenocarcinoma	potentially promotes immune cell infiltration	(113)
	TET3	/	possibly promotes prostate cancer	/	(38)
	ALKBH1	tRNA, mRNA	converts m^5^C into hm^5^C	decreases m^5^C levels	(109)
		tRNA^Leu-CAA^ (C34)	converts m^5^C into hm^5^C or f^5^C	promotes the decoding of Leu codons under stress	(109, 114)
		tRNA^Met^ (C34)	converts m^5^C into f^5^C	promotes the translation of AUA, a non-universal codon in mammalian mitochondria which is significant for mitochondrial functions	(114)

ETS, external transcribed sequence; snoRNA, small nucleolar RNA; Tat, transactivator; TAR RNA, transactivation response RNA; GRB2, growth factor receptor-bound protein 2; PIK3R1, phosphoinositide-3-kinase regulatory subunit 1; PCYT1A, phosphate cytidylyltransferase 1 choline-alpha; FOXC2, Forkhead box protein C2; p57^Kip2^, the cyclin-dependent kinase (CDK) inhibitor; LIN28B, protein lin-28 homolog B; PI3K/AKT, phosphatidylinositol 3-kinase/protein kinase B; ERK/MAPK, extracellular-signal-regulated kinases/mitogen-activated protein kinases; TEAD1, first member of TEA/ATTS domain transcription factor family; AR, androgen receptor; KRT13, keratin 13; YBX1, Y-box binding protein 1; TME, tumor microenvironment; TIGAR, TP53-induced glycolysis and apoptosis regulator; MTERF4, mitochondrial transcription termination factor 4; SOX9, SRY-box transcription factor 9; CDK10, cyclin-dependent kinases 10; PGC-1α, peroxisome proliferator-activated receptor-gamma coactivator 1 alpha; eIF4A3, eukaryotic translation initiation factor 4A3; USP3, ubiquitin specific peptidase 3; YAP, Yes-associated protein; PKM2, pyruvate kinase M2; NEAT1, nuclear enriched abundant transcript 1; YBX2, Y-box-binding protein 2; CDKN1A, cyclin-dependent kinase inhibitor 1A; SMO, smoothened; HDGF, heparin binding growth factor; ELAVL1, ELAV-like RNA binding protein 1; NKILA, NF-kappa B interacting lncRNA; PEBR1, phosphatidylethanolamine binding protein 1; Pabpc1a, poly A binding protein cytoplasmic 1a; hm^5^C, 5-hydroxymethylcytosine; f^5^C, 5-formylcytidine.

### Writers

To date, the m^5^C methylation of RNA, including mRNA, rRNA and tRNA, is believed to be mainly mediated by two groups of RNA methyltransferases, DNMT2 and the NSUN protein family.

DNMT2, also known as TRDMT1 (tRNA methyltransferase 1), generally influences tRNA methylation (25). The m^5^C site is located on cytosine 38 in the anticodon loop of tRNA^Asp-GUC^, tRNA^Gly-GCC^, tRNA^Val-AAC^ (39, 40), and receives a methyl group from the cofactor S-adenosyl-methionine (SAM) (115). Studies showed that simultaneous knockout of DNMT2 and NSUN2 led to deficient tRNA methylation, protein synthesis and cellular differentiation, causing the death of experimental mice, although deficiency of either DNMT2 or NSUN2 alone did not show detectable effects. The results suggested that DNMT2 plays a role in tRNA methylation and cell survival (41). In addition, the role of DNMT2 in mRNA methylation and expression modulation was also reported. DNMT2 deficiency is associated with alterations in mRNA expression and methylation profiles and the inhibition of cell proliferation and migration (42).

The NSUN family also utilizes SAM as a methyl donor (116). The NSUN family consists of seven members, NSUN1-7, and each target different types of RNAs. The RNA targeting specificity of the NSUN family was reviewed in 2019 by Katherine E. Bohnsack et al., with NSUN1, 4, and 5 responsible for rRNA methylation, NSUN2, 3, and 6 responsible for tRNA methylation (NSUN2 also promotes mRNA methylation), and NUSN7 responsible for enhancer RNA (eRNA) methylation (25). However, within the last three years, studies have provided new insights in this regard. Among the seven RNA methyltransferases, NSUN2 was the first discovered and most widely studied. The role of NSUN2 in promoting tRNA methylation is wellknown, and NSUN2 deficiency directly causes a decrease in tRNA m^5^C levels (47). Notably, NSUN2 is predominantly distributed in the nucleus and catalyzes methylation of cytoplasmic tRNA; it is also capable of introducing m^5^C to mitochondrial tRNA (48, 49). However, NSUN2 silencing did not significantly affect mitochondrial tRNA stability (49), which suggested that NSUN2 may not be necessary for tRNA methylation within the mitochondria. NSUN2 is also responsible for biological processes, including cell proliferation (50) and carcinogenesis. Enhanced levels of NSUN2 and NSUN2-mediated m^5^C are observed in patients with gastric cancer (GC) (30, 34, 51), esophageal squamous cell carcinoma (ESCC) (52, 53), hepatocellular carcinoma (HCC) (35, 54), hypopharyngeal squamous cell carcinoma (HPSCC) (55), prostate cancer (56), cervical cancer (57), nasopharyngeal carcinoma (58) and uveal melanoma (59). NSUN2 also affects immune cells, as hyperexpression of NSUN2 in T cells promotes IL-17A secretion by methylating IL-17A mRNA at cytosine C466 both *in vitro* and *in vivo*, which stimulates its translation (60). In addition, under conditions of oxidative stress-induced cellular senescence, NSUN2-mediated m^5^C, together with METTL3/METTL14-mediated m^6^A, synergistically upregulates the expression of p21 (61). Note that NSUN2 is also involved in the functioning of m^5^C readers, including ALYREF, whose nuclear-cytoplasm transportation is partly modulated by NSUN2 (62). Other members of the NSUN family are also active in catalyzing m^5^C RNA modifications. NSUN1, or NOP2 (nucleolar protein 2), catalyzes rRNA m^5^C modifications, thus affecting biological processes including ribosome biogenesis (43), cell proliferation (44), healthspan modulation (45) and HIV-1 viral latency (46). NSUN3, a putative tRNA methyltransferase, plays a role in tumor progression (27, 63, 64), immune cell infiltration (64, 66) and multisystem mitochondrial diseases (67). Mechanistically, m^5^C modification of tRNA occurs at C34 in the anticodon loop (65, 117). Current studies have revealed that NSUN3 expression is upregulated in patients with low-grade glioma (63) and head and neck squamous cell carcinoma (HNSCC) (64), and NSUN3-mediated m^5^C modification of tRNA enhances metastasis by stimulating the translation of mitochondrial mRNA (27). NSUN3-associated immune cell infiltration mainly includes CD8+ T cells (66) and M2 macrophages (64). Deficiency of NSUN3 also leads to severe dysfunction within the mitochondria, such as combined oxidative phosphorylation deficiency, which may lead to early-onset encephalomyopathy and seizures (67). NSUN4 facilitates mitoribosomal assembly by methylating C911 in 12S rRNA and interacting with MTERF4 (mitochondrial transcription termination factor 4) (68). In addition to rRNA methylating activity, NSUN4 also acts as a tRNA (69) and mRNA (70) methyltransferase, and NSUN4-mediated m^5^C modification in the 3’-UTR (3’-untranslated region) of SOX9 (SRY-box transcription factor 9) mRNA is necessary for adaptation to higher temperatures (69) and chondrogenic differentiation regulated by SOX9 (70). NSUN4 also promotes HCC generation (71, 72) and neutrophil infiltration (66). NSUN5 participates in rRNA methylation, introducing m^5^C3782 into human and m^5^C3438 into mouse 28S rRNA (73, 74). Overexpression of NSUN5 is associated with tumorigenesis in HCC (75) and colorectal cancer (CRC) patients (76), while NSUN5 deficiency causes a reduction in total protein synthesis, thus impairing cell proliferation (73). In patients with tetralogy of Fallot (TOF) (118) and William’s-Beuren syndrome (WBS) (119), NSUN5 is drastically downregulated. Previous studies regarded NSUN6 as at RNA methyltransferase, which identifies C72 at the 3′ end of the tRNA acceptor stem and targets tRNA^Cys^ and tRNA^Thr^ (77, 78), but recent investigations have discovered that NSUN6 exhibits mRNA methylating bioactivity (79, 82, 120, 121). mRNA methylated by NSUN6, which primarily targets the 3’-UTR at the consensus sequence motif CTCCA, increased in transcript and protein levels (79). The role of NSUN6 in cancer development remains unclear, but studies have shown that NSUN6 acts as a protective factor against triple-negative breast cancer (TNBC) (36), pancreatic cancer (31), testis cancer (79), thyroid cancer (79) and ovary cancer (79) but is a risk factor for CRC (80, 81). One possible explanation of the controversial role of NSUN6 in different types of cancers is that NSUN6 expression level in different immune cells within the TME differs based on the tumor context. For instance, NSUN6 is mainly expressed in Tregs in TNBC (36), but in exhausted CD8+ T cells, proliferating T cells and myofibroblasts in CRC (80). In addition, NSUN6 is also related to the promotion of the cell cycle (82), infiltration of B cells, CD4+ T cells and CD8+ T cells (36, 80), and formation of antibody-secreting plasma cells (83). NSUN7 methylates eRNA, a noncoding RNA associated with transcription modulation, and enhances the expression of mRNAs coding for Pfkl, Sirt5, Idh3b and Hmox2 in a peroxisome proliferator-activated receptor-gamma coactivator 1 alpha (PGC-1α)-dependent manner. These effects are likely to facilitate adaptive metabolic alterations under starvation (84).

### Readers

Readers, or binding proteins of m^5^C sites, include ALYREF and YBX1. ALYREF is reported to be an important oncogenic factor and is associated with poor prognosis in patients with various types of cancer, including HCC (72, 85, 86), glioblastoma (87, 88), glioma (63), neuroblastoma (89), lung adenocarcinoma (90, 91), HNSCC (92, 93), bladder cancer (32) and breast cancer (94, 95). For example, elevated levels of ALYREF in HCC patients were found to be responsible for upregulated eIF4A3 expression (86) and abnormal cell cycle and mitosis (72). In lung adenocarcinoma patients, ALYREF, together with NSUN2, promotes m^5^C modification of YAP (Yes-Associated Protein) mRNA in the 3’-UTR, thus increasing the stability of YAP mRNA and causing enhanced exosome secretion, tumor malignancy and drug resistance (91). In contrast, ALYREF is considered a protective factor against colon adenocarcinoma (81), but the mechanism remains to be elucidated. In addition, ALYREF also participates in the regulation of adipogenesis (96), myogenesis (96, 97) and retrovirus replication (98) and may have biological activity in the context of abdominal aortic aneurysm (AAA) (99).

YBX1 is another m^5^C reader that has multiple functions in cancer development and embryo development. Oncogenic effects of YBX1 are found in GC (51), bladder cancer (100), glioblastoma (101), CRC (76, 102), cholangiocarcinoma (103), clear cell renal cell carcinoma (ccRCC) (104), prostate cancer (56), epithelial ovarian cancer (105) and cervical cancer (57), mainly owing to YBX1-RNA interactions that have stabilizing effects on target RNAs. For example, in GC patients, YBX1 binds with FOXC2 (Forkhead box protein C2) mRNA, which is m^5^C-modulated by NSUN2, to enhance its tumor-promoting ability (51). In bladder cancer patients, YBX1 stabilizes oncogenic HDGF (heparin binding growth factor) mRNA by targeting the m^5^C-modified site on its 3′-UTR (100). Interestingly, in addition to its oncogenic effects, YBX1 is also important in normal cell proliferation and embryo development. For instance, animal experiments revealed that YBX1 is essential for embryonic brain development in mice (101), and YBX1 deficiency causes early gastrulation defects in zebrafish embryos (106).

### Erasers

Currently discovered m^5^C demethyltransferases are the TET family and ALKBH1. The TET family is a group of Fe(II) and alpha-ketoglutarate-dependent m^5^C dioxygenases that convert 5-methylcytosine (m^5^C) to 5-hydroxymethylcytosine (hm^5^C) (122). These enzymes were originally discovered in the translocation breakpoint of t(10;11) in patients with infant acute myeloid leukemia (AML), hence the name (123). Overall, the scarcity of research focusing on m^5^C erasers limits the comprehensive understanding of these proteins. TET1-mediated mRNA m^5^C demethylation is essential for completion of DNA repair and survival of cells in the context of DNA damage (107). TET2 mainly facilitates the conversion of m^5^C into hm^5^C, thus leading to the elimination of m^5^C modification in RNA (108, 109), but the effects are not as strong as ALKBH1 (109). TET2 expression was measured in patients with various types of cancers, and it was upregulated in low-grade glioma patients (63) but downregulated in ccRCC (110, 111), ovarian cancer (112) and prostate adenocarcinoma (113) patients. Note that the potential tumor-suppressive effect of TET2 in prostate adenocarcinoma is likely to be linked with enhanced immune cell infiltration (113). TET3-mediated m^5^C elimination has not been clarified to date. However, some researchers have reported that upregulated TET3 expression in prostate cancer patients might be associated with poor prognosis (38).

ALKBH1 has been identified as a demethyltransferase for both RNA and DNA (124), but most studies focused on ALKBH1-demethylated DNA modifications, with fewer researchers concentrating on the RNA part. So far, studies have revealed that ALKBH1 takes part in the transformation of m^5^C RNA modifications to either hm^5^C or f^5^C (5-formylcytidine) RNA modifications (109, 114). More specifically, in cytoplasmic tRNA, ALKBH1 targets the wobble position (position 34) of tRNA^Leu-CAA^ and converts m^5^C RNA modifications to hm^5^C or f^5^C, promoting the decoding of Leu codons under stress (109, 114). At the same position (position 34) of mitochondrial tRNA^Met^, only the alteration from m^5^C to f^5^C was found, which was proved indispensable for the translation of AUA, a non-universal codon in mammalian mitochondria, indicating that ALKBH1-mediated m^5^C RNA modification removal is significant for mammalian mitochondrial functions (114). Interestingly, an *in vitro* experiment showed that ALKBH1 first hydroxylates m^5^C to form hm^5^C, and then oxidizes hm^5^C to form f^5^C, meaning ALKBH1-mediated biogenesis of hm^5^C and f^5^C is actually two relevant and coherent processes (114).

## m^5^C RNA modification in cancer cells

**Figure 4:**
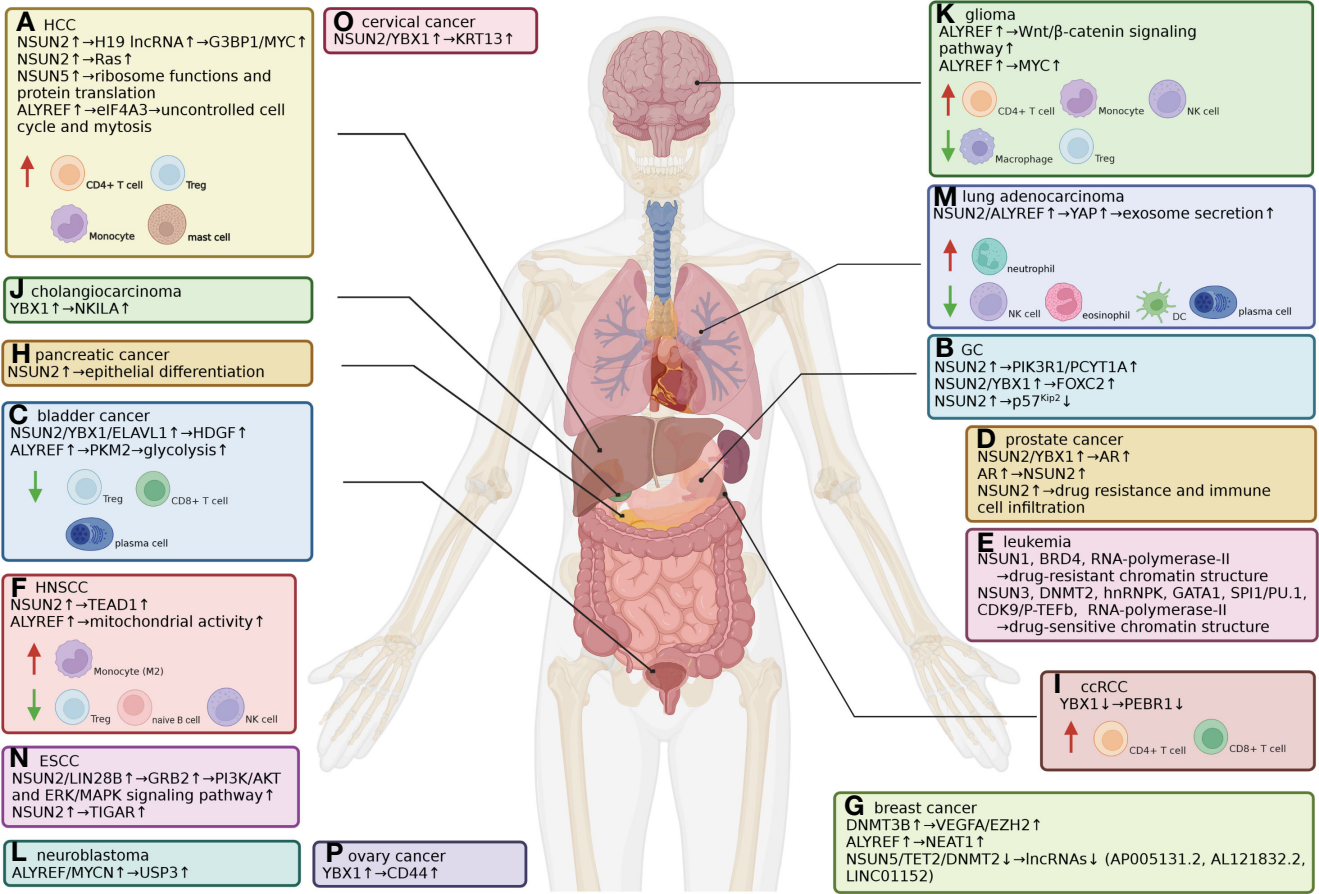
Expression of m^5^C-related genes and immune cell infiltration in different cancer types. **(A)** HCC. NSUN2-mediated m^5^C modulation of H19 lncRNA increases its stability, leading to enhanced recruitment of G3BP1 and MYC. NSUN2 also promotes HCC progression by modulating the Ras signaling pathway, the cell cycle and drug resistance. NSUN5 facilitates ribosomal functions and protein translation. ALYREF upregulates eIF4A3 expression, which leads to uncontrolled mitosis. The abundance of CD4+ T cells (including Tregs), M0, M1 and M2 macrophages and resting mast cells is higher in HCC tissues from patients with poor prognoses. **(B)** GC. NSUN2 methylates PIK3R1 and PCYT1A mRNA, stabilizing them and activating downstream cancerous signaling pathways. NSUN2 methylates FOXC2 mRNA, enhancing its interaction with the m^5^C reader YBX1. NSUN2 destabilizes tumor-suppressive p57Kip2 mRNA by m^5^C-methylation in its 3’-UTR. **(C)** Bladder cancer. ALYREF binds to the 3’-UTR of PKM2 mRNA, stabilizing it and enhancing PKM2-mediated glycolysis. NSUN2 mediates m^5^C modification in the 3’-UTR of oncogenic HDGF mRNA; YBX1 recruits ELAVL1 to form a m^5^C-binding complex to stabilize HDGF mRNA. Lower Treg, CD8+ T-cell and plasma cell infiltration rates indicate poor prognosis. **(D)** Prostate cancer. NSUN2 catalyzes and YBX1 recognizes m^5^C modification sites on androgen receptor (AR) mRNA, and AR positively regulates NSUN2 transcription in return. NSUN2 expression also leads to drug resistance and immune cell infiltration. **(E)** Leukemia. NSUN1 forms an active drug-resistant chromatin structure with BRD4 and RNA polymerase-II, while SUN3 and DNMT2 form a drug-sensitive structure with hnRNPK, GATA1, SPI1/PU.1, and CDK9/P-TEFb to recruit RNA polymerase-II. **(F)** HNSCC. NSUN2 promotes HNSCC by suppressing immune infiltration and methylates and stabilizes TEAD1 mRNA. ALYREF increases mitochondrial activity to ensure tumor cells are supplied with energy. Lower Treg, naïve B-cell and NK cell infiltration indicates poor prognosis, while higher M2 macrophage infiltration indicates poor prognosis. **(G)** Breast cancer. ALYREF promotes breast cancer by enhancing the transcription of NEAT1 lncRNA. DNMT3B targets VEGFA and EZH2 as tumor promoters. NSUN5, TET2 and DNMT2 exert inhibitory effects on breast cancer by modifying three lncRNAs. **(H)** Pancreatic cancer. NSUN2 promotes pancreatic cancer and epithelial differentiation. **(I)** ccRCC. YBX1 negatively modulates ccRCC by binding and stabilizing PEBR1 mRNA. The abundance of CD4+ T cells and CD8+ T cells was higher in ccRCC tissues. **(J)** Cholangiocarcinoma. YBX1 promotes tumor development by stabilizing m^5^C-methylated NKILA. **(K)** Glioma. ALYREF activates the Wnt/β-catenin signaling pathway and stabilizes MYC mRNA, promoting the development of glioblastoma, a malignant type of glioma. The infiltration of CD4+ T cells, monocytes and NK cells decreases in glioma tissues, while macrophage and Treg infiltration increases. **(L)** Neuroblastoma. ALYREF forms a nuclear coactivator complex with MYCN to stimulate USP3 transcription, which promotes tumorigenesis. **(M)** Lung adenocarcinoma. SUN2 and ALYREF increase YAP mRNA stability, thus enhancing exosome secretion, tumor malignancy and drug resistance. Lower plasma cell, eosinophil, NK cell and DC infiltration rates and higher neutrophil infiltration rates indicate poor prognosis. **(N)** ESCC. NSUN2 methylates GRB2 in a LIN28B-dependent manner, thus activating the PI3K/AKT and ERK/MAPK signaling pathways. NSUN2 also promotes TIGAR to enhance tumor growth. **(O)** Cervical cancer. NSUN2 and YBX1 promote cervical cancer by increasing the expression levels of KRT13 mRNA. **(P)** Ovarian cancer. YBX1 modulates CD44 expression to enhance chemoresistance.

As discussed above, RNA m^5^C modification has been discovered to be an important biological process in many types of diseases, including cancer. Dysfunction or alterations in the expression levels of m^5^C writers, readers and erasers influence tumor development, malignancy and metastasis by changing both mRNA and noncoding RNAs at the expressional and transcriptional levels (**Figure 4**). Here, we present a detailed overview of m^5^C-mediated alterations within tumor cells that have been clarified to date (**Table 2**). Understanding the molecular mechanisms of m^5^C-mediated tumorigenesis is vastly important for increasing the therapeutic efficiency of antitumor treatments.

**Table 2:** Expression of m^5^C-related genes and tumor-promoting/suppressing mechanisms in different types of tumors.

Cancer Types	Related Enzymes	Expression	Target RNAs	Effects	Mechanisms	References
HCC	NSUN2	upregulated	H19 lncRNA	tumor-promoting	m^5^C-methylates H19 lncRNA, leading to MYC stimulation	(35)
			/	tumor-promoting	modulates Ras signaling pathway and cell cycles, causing drug resistance	(54)
	NSUN4	upregulated	/	tumor-promoting	/	(71)
	NSUN5	upregulated	/	tumor-promoting	facilitates ribosome functions and protein translation	(75)
	ALYREF	upregulated	/	tumor-promoting	causes uncontrolled cell cycle and mitosis	(72)
			/	tumor-promoting	stimulates eIF4A3 expression	(86)
	/	/	circRNA	tumor-promoting	/	(125)
GC	NSUN2	upregulated	PIK3R1 and PCYT1A mRNA	tumor-promoting	m^5^C-methylates PIK3R1 and PCYT1A mRNA, stabilizing them and activating downstream cancerous signaling pathways	(34)
			FOXC2 mRNA	tumor-promoting	m^5^C-methylates FOXC2 mRNA, enhancing its interaction with m^5^C reader YBX1	(51)
			p57^Kip2^ mRNA	tumor-promoting	destabilizes tumor-suppressive p57Kip2 mRNA by m^5^C-methylation in its 3’-UTR	(30)
	/	/	/	tumor-promoting	immune suppression	(126)
bladder cancer	NSUN2, YBX1	upregulated	HDGF mRNA	tumor-promoting	NSUN2 mediates m^5^C modification in 3’-UTR of oncogenic HDGF mRNA; YBX1 recruits ELAVL1 and together forms a m^5^C-binding complex to stabilize HDGF mRNA	(100)
	ALYREF	upregulated	PKM2 mRNA	tumor-promoting	stabilizes PKM2 mRNA by binding to its 3’-UTR, enhancing PKM2-mediated glycolysis	(32)
prostate cancer	NSUN2, YBX1	upregulated	AR mRNA	tumor-promoting	NSUN2 reciprocally increasesAR translation *via* m^5^C-modulating AR mRNA in a YBX1-dependent manner	(56)
	NSUN2	upregulated	/	tumor-promoting	affects drug resistance and immune cell infiltration	(37)
	TET3	upregulated	/	tumor-promoting	/	(38)
leukemia	NSUN1	/	/	tumor-promoting	forms an active chromatin structure with BRD4 and RNA-polymerase-II, which responds poorly to 5-AZA treatment	(127)
	NSUN3, DNMT2	/	/	tumor-suppressive	bind directly with hnRNPK which interacts with GATA1, SPI1/PU.1 and CDK9/P-TEFb to recruit RNA-polymerase-II at precursor RNA, forming chromatin structures that are sensitive to 5-AZA treatment	(127)
HNSCC	NSUN3	upregulated	/	tumor-promoting	promotes tumor progression by regulating immune infiltration	(64)
	NSUN2	upregulated	/	tumor-promoting	negatively regulates immune cell infiltration in TME, promoting nasopharyngeal carcinoma (NPC)	(58)
		upregulated	TEAD1 mRNA	tumor-promoting	m^5^C-methylates oncogenic TEAD1 mRNA and upregulates its expression level, promotes hypopharyngeal squamous cell carcinoma (HPSCC)	(55)
	ALYREF	upregulated	/	tumor-promoting	enhances mitochondrial activity and intracellular energy metabolism, which ensures continuous energy supplies for timorous tissues	(92)
breast cancer	NSUN2	upregulated	/	tumor-promoting	/	(36)
	ALYREF	upregulated	NEAT1 lncRNA	tumor-promoting	binds with oncogenic NEAT1 lncRNA promoter region, enhancing its transcription	(94)
	DNMT3B	upregulated	/	tumor-promoting	targets VEGFA and EZH2	(95)
	NSUN6	downregulated	/	tumor-suppressive	/	(36)
	NSUN5, TET2, DNMT2	downregulated	lncRNA	tumor-suppressive	modifies three lncRNAs	(128)
pancreatic cancer	NSUN2	upregulated	mRNA	tumor-promoting	regulates pancreatic tumorigenesis and epithelial differentiation through mRNA methylation	(129)
	/	/	/	tumor-promoting	m^5^C modification causes immune evasion and enhances PD-L1 expression	(130)
	NSUN6, DNMT3A	downregulated	/	tumor-suppressive	/	(31, 131)
ccRCC	DNMT3B, NSUN1, NSUN2, NSUN5	upregulated	/	tumor-promoting	/	(110, 111, 132)
	NSUN6, TET2	downregulated	/	tumor-suppressive	/	(111)
	YBX1	downregulated	PEBR1 mRNA	tumor-suppressive	YBX1/EVAVL1 complex binds and stabilizes PEBR1 mRNA, which negatively modulates ccRCC	(104)
CRC	NSUN5, NSUN6, ALYREF, YBX1	upregulated	/	tumor-promoting	/	(76, 81)
	/	/	/	tumor-promoting	inhibits tumor infiltration of immune cells	(133)
cholangiocarcinoma	YBX1	upregulated	NKILA	tumor-promoting	recognizes and stabilizes m^5^C-methylated NKILA	(103)
glioma	NSUN1-5, NSUN7, DNMT1, DNMT3B, YBX-1	upregulated	/	tumor-promoting	/	(63, 101, 134)
	NSUN6	downregulated	/	tumor-suppressive	/	(63, 134)
glioblastoma	ALYREF	upregulated	/	tumor-promoting	activates Wnt/β-catenin signaling pathway and reciprocally stabilizes MYC mRNA	(87, 88)
neuroblastoma	ALYREF	upregulated	/	tumor-promoting	forms a nuclear coactivator complex with MYCN to stimulate USP3 transcription	(89)
lung adenocarcinoma	NSUN2, ALYREF	upregulated	YAP mRNA	tumor-promoting	increase YAP mRNA stability, thus enhancing exosome secretion, tumor malignancy and drug resistance	(91)
ESCC	NSUN2	upregulated	GRB2 mRNA	tumor-promoting	m^5^C-methylates GRB2 *via* LIN28B-dependent way, thus activating PI3K/AKT and ERK/MAPK signaling pathway; promotes TIGAR	(52, 53)
cervical cancer	NSUN2, YBX1	upregulated	KRT13 mRNA	tumor-promoting	promote KRT13 mRNA methylation and translational activation	(57)
ovarian cancer	YBX1	upregulated	/	tumor-promoting	YBX1 modulates the expression of a variety of downstream targets, including CD44, thus enhancing chemoresistance	(105)
	NSUN6	downregulated	/	tumor-suppressive	/	(79)
testis cancer	NSUN6	downregulated	/	tumor-suppressive	/	(79)
thyroid cancer	NSUN6	downregulated	/	tumor-suppressive	/	(79)
uveal melanoma	NSUN2	upregulated	/	tumor-promoting	/	(59)

VEGFA, vascular endothelial growth factor A; EZH2, enhancer of zeste homolog 2; BRD4, bromodomain-containing protein 4; 5-AZA, 5-azacitidine; hnRNPK (heterogeneous nuclear ribonucleoprotein K; GATA1, GATA binding protein 1; SPI1/PU.1, recombinant spleen focus forming virus proviral integration 1/purine rich box-1; CDK9/P-TEFb, cyclin-dependent kinase 9/positive transcription elongation factor b.

### Hepatocellular carcinoma (HCC)

Previous studies have demonstrated a clear relationship between high m^5^C levels and HCC development, migration and malignancy. Recent studies have mainly focused on lncRNAs. For example, during HCC, the expression levels of m^5^C-associated genes, including NSUN2 (35, 54), NSUN4 (71), NSUN5 (75) and ALYREF (86), increase. NSUN2-mediated m^5^C modulation of H19 lncRNA increases its stability, leading to enhanced recruitment of the G3BP1 (Ras-GTPase-activating protein-binding protein 1) oncoprotein, a potential enhancer of MYC accumulation (35). NSUN2 also modulates the Ras signaling pathway as well as the cell cycle, thus allowing for tumor escape from chemotherapy (54). A bioinformatics analysis discovered that NSUN5 overexpression was positively associated with enhanced ribosome functions and protein translation within HCC cells (75). ALYREF dysfunction is responsible for aberrant cell cycle regulation and mitosis of HCC cells (72) and promotes HCC possibly *via* stimulation of eIF4A3 expression (86). Thus, suppressors for ALYREF and eIF4A3, such as miR-4666a-5p and miR-6124, are promising therapeutic agents (86). Moreover, m^5^C modulation of circRNA is also important in HCC development (125). In addition, alterations in the tumor microenvironment (TME) and immune cell infiltration also contribute to m^5^C-mediated HCC development (135, 136). Currently, researchers are investigating new methods for prognosis prediction in HCC patients and constructed speculating models based on m^5^C-related modulators, such as the NSUN family, TET1, and YBX1 (72, 137, 138). These findings may have profound clinical implications.

### Gastric cancer (GC)

The role of m^5^C RNA modification in GC is generally oncogenic, with high levels of m^5^C indicating poor prognosis and a low overall survival (OS) rate (126). Modifications of both mRNA (30, 34, 51) and lncRNA (126, 139) have been observed during GC progression, and risk models based on m^5^C levels were developed for prognosis prediction (139). NSUN2 is the main oncogenic m^5^C-methyltransferase in GC, targeting the mRNAs of PIK3R1 (phosphoinositide-3-kinase regulatory subunit 1) (34), PCYT1A (phosphate cytidylyltransferase 1 choline-alpha) (34), FOXC2 (Forkhead box protein C2) (51) and p57^Kip2^ (a type of cyclin-dependent kinase (CDK) inhibitor) (30). After m^5^C modulation and binding to m^5^C readers, such as YBX1, the transcriptional activity of PIK3R1, PCYT1A and FOXC2 mRNA was increased, while tumor-suppressive p57^Kip2^ mRNA was destabilized as a result of m^5^C modulation in the 3’-UTR. Consequently, the elevated NSUN2 levels in GC patients lead to enhanced proliferation, migration, and invasion of cancerous cells. NSUN2 activators, such as small ubiquitin-like modifier (SUMO)-2/3, which directly interact with NSUN2 to stabilize and mediate its nuclear transport, promote the development of GC (34). The oncogenic interaction between NSUN2 and FOXC2 mRNA can be facilitated by lncRNA FOXC2-AS1 (FOXC2 antisense RNA 1) (51). The oncogenic role of m^5^C modulation may also be linked to immune suppression, as patients with lower levels of m^5^C modulation were found to have higher levels of immune activation and longer progression-free survival (PFS) and OS (126).

### Bladder cancer

m^5^C-mediated cell proliferation is considered one of causes of bladder cell malignancy. Overexpressed ALYREF in bladder cancer cells interacts with the 3’-UTR of PKM2 (pyruvate kinase M2) mRNA, causing its stabilization and enhanced PKM2-associated glycolysis (32). ALYREF stimulators, such as hypoxia‐inducible factor‐1alpha (HIF‐1α), significantly increase the expression levels of ALYREF and PKM2 and are correlated with poor prognosis (32). Another m^5^C reader, YBX1,maintains the stability of its target mRNA, oncogenic HDGF mRNA methylated by NSUN2, by forming a 3’-UTR-binding complex with ELAVL1 (ELAV like RNA binding protein 1) (100). Moreover, high expression of immune cells, including regulatory T cells (Tregs), CD8+ T cells, plasma cells and activated dendritic cells, is related to a good prognosis, while high expression of resting CD4+ memory T cells, M0 macrophages, M1 macrophages, M2 macrophages and neutrophils show the opposite trend (140).

### Prostate cancer

Several prognostic models based on m^5^C modulators (38) or m^5^C-related lncRNAs (141) have been developed for prostate cancer patients. Specifically, experimental results showed that increased NSUN2 (56), YBX1 (56) and TET3 (38) levels correlate with a poor prognosis. Posttranscriptional m^5^C modification of androgen receptor (AR) mRNA by NSUN2 is recognized by YBX1, increasing AR mRNA stability and translation (56). Interestingly, AR positively regulates NSUN2 at the transcriptional level (56), forming a reciprocal activation loop. High NSUN2 expression is also associated with low chemotherapeutic sensitivity and immune cell infiltration (37). In addition to NSUN2, immune cell infiltration characteristics are associated with many other m^5^C regulators, such as NSUN6 and TET1-3 (38, 113).

### Leukemia

RNA m^5^C modifications affect tumor malignancy and drug resistance not only in solid tumors but also in nonparenchymal tumors, including leukemia. In 2018, David G. Courtney et al. determined that NSUN1 is partly responsible for the formation of the 5-AZA (5-azacitidine)-insensitive chromatin structure during leukemia, which causes drug resistance (127). Mechanistically, NSUN1 forms an active chromatin structure with BRD4 (bromodomain-containing protein 4) and RNA-polymerase-II, which responds poorly to 5-AZA but well to the BRD4 inhibitor JQ1 or miRNA targeting NSUN1. In contrast, another two m^5^C regulators, NSUN3 and DNMT2, bind directly with heterogeneous nuclear ribonucleoprotein K (hnRNPK), a conserved RNA-binding protein that interacts with the lineage-determining transcription factors GATA binding protein 1 (GATA1), recombinant spleen focus forming virus proviral integration 1/purine rich box-1 (SPI1/PU.1) and cyclin-dependent kinase 9 (CDK9)/positive transcription elongation factor b (P-TEFb) to recruit RNA-polymerase-II to RNA precursors, forming chromatin structures that are sensitive to 5-AZA (127).

### Head and neck squamous cell carcinoma (HNSCC)

HNSCC refers to a group of epithelium-derived cancers that occur in the mucosal surfaces of the head and neck, including the oral and nasal cavity, oropharynx, nasopharynx, larynx and hypopharynx. To date, statistics have revealed that almost all m^5^C regulators show elevated expression levels during HNSCC, with the exception of NSUN7 and TET2 (93, 142, 143), suggesting that they play different roles in HNSCC tumorigenesis. Mechanistically, NSUN3 promotes tumor progression by regulating immune infiltration (64), and ALYREF enhances mitochondrial activity and intracellular energy metabolism, which ensures continuous energy supplies for timorous tissues (92). In nasopharyngeal carcinoma (NPC) specifically, NSUN2 negatively regulates immune cell infiltration in the TME (58). In addition, NSUN2 promotes hypopharyngeal squamous cell carcinoma (HPSCC) by m^5^C-methylating oncogenic TEAD1 (TEA domain transcription factor 1) mRNA, which upregulates its expression level (55).

### Breast cancer

m^5^C RNA modification has dual effects on breast cancer development. Recent studies suggest NSUN2 (36), ALYREF (94, 95) and DNMT3B (95) as risk factors, while NSUN5 (128), NSUN6 (36), TET2 (128), and DNMT2 (128)are protective factors. Mechanistically, ALYREF, which was found amplified both at the mRNA and protein levels, binds with the oncogenic NEAT1 lncRNA promoter region, enhancing its transcription (94). Additionally, enrichment analysis revealed that vascular endothelial growth factor A (VEGFA) and enhancer of zeste homolog 2 (EZH2) were potential targets of DNMT3B (95). In addition, NSUN5, TET2, and DNMT2 modified three lncRNAs, namely, AP005131.2, AL121832.2, and LINC01152, to be protective factors against breast cancer (128).

### Pancreatic cancer

As in many other types of cancers, NSUN2 regulates pancreatic tumorigenesis and epithelial differentiation through mRNA methylation (129). In contrast, NSUN6 (31) and DNMT3A (131) have inhibitory effects on pancreatic cancer and suppress the proliferation of cancerous cells, but the mechanisms remain to be elucidated. m^5^C modification profoundly influences the tumor immune microenvironment (130, 144), interfering with the infiltration of CD8+ T cells and upregulating PD-L1 expression (130). Risk models based on m^5^C-related lncRNAs have also been constructed to provide prognostic information (145).

### Clear cell renal cell carcinoma (ccRCC)

m^5^C modification has dual effects on ccRCC development. YBX1 negatively modulates ccRCC by binding and stabilizing PEBR1 mRNA, a tumor suppressor gene (104). Other m^5^C-related genes, such as DNMT3B, NSUN1, NSUN2 and NSUN5, are highly expressed in ccRCC patients and correlate with worse prognosis (110, 111, 132), while NSUN6 and TET2 mainly function as protective factors (111). Notably, the role of NSUN4 in ccRCC remains controversial, as studies have obtained opposing results (110, 111).

### Other cancer types

In CRC patients, increased levels of m^5^C-related regulators, such as NSUN5, NSUN6, ALYREF and YBX1, were found (76, 81). The m^5^C levels of peripheral blood immune cells showed higher CRC diagnostic value than that of common blood tumor biomarkers (76), which is correlated with the discovery that m^5^C modification inhibits tumor infiltration of immune cells (133). In cholangiocarcinoma patients, the m^5^C-modified functional lncRNA NKILA (NF-kappa B interacting lncRNA), which is recognized and stabilized by YBX1, is associated with advanced TNM stage and poor prognosis (103). In glioma patients, m^5^C-associated genes, including NSUN1-5, NSUN7, DNMT1, DNMT3B and YBX-1, are upregulated, with the exception of NSUN6 (63, 101, 134). In patients with glioblastoma, the most aggressive diffuse glioma, upregulated ALYREF plays an oncogenic role by activating the Wnt/β-catenin signaling pathway and stabilizing MYC mRNA (87, 88). Interestingly, MYC also exerts positive impacts on ALYREF, forming a positive feedback loop (87). In neuroblastoma patients, the m^5^C reader ALYREF forms a nuclear coactivator complex with MYCN to stimulate USP3 transcription, which promotes the tumorigenesis of neuroblastoma (89). In lung adenocarcinoma patients, NSUN2 and ALYREF were found to be oncogenic through interacting with YAP mRNA. The m^5^C modification in the 328-331 3’-UTR of YAP mRNA increases its stability, enhances exosome secretion, and stimulates the transcription of seven downstream exosome-promoting genes. Together, m^5^C-mediated YAP stimulation leads to increased tumor malignancy and drug resistance (91). In addition, risk models based on m^5^C regulators or m^5^C-related lncRNAs were also developed for prognosis prediction (11, 90, 146). In patients with ESCC, NSUN2 promotes ESCC progression and chemoresistance by promoting TIGAR (TP53 induced glycolysis regulatory phosphatase) (53) and GRB2 (growth factor receptor bound protein 2) (52). The positive influence on GRB2 is achieved by NSUN2-mediated LIN28B-dependent m^5^C modification of GRB2 mRNA, which indirectly activates the PI3K/AKT and ERK/MAPK signaling pathways (52). In cervical cancer patients, NSUN2 and YBX1, which catalyze and recognize methylation sites, respectively, induce KRT13 mRNA methylation and translational activation (57). In ovarian cancer patients, YBX1 modulates the expression of a variety of downstream targets, including CD44, thus enhancing chemoresistance (105). In contrast, RNA m^5^C modification mediated by the methyltransferase NSUN6 suppresses testis, thyroid and ovary cancers (79). Finally, NSUN2-mediated RNA m^5^C modification modulates uveal melanoma cell proliferation and migration, although the exact mechanisms remain unknown (59).

## m^5^C RNA modification in immune cells

Current studies have revealed that most immune cells, including T cells from different subgroups (e.g., CD4+ T cells, CD8+ T cells, Tregs), B cells and plasma cells, NK cells, NKT cells, macrophages, granulocytes and mast cells, manifest alterations in cell expression, infiltration and recruitment rate, which is concluded by several prognostic models constructed based on m^5^C-related lncRNAs (147), m^5^C-regulated genes (38, 148) or m^5^C-related differentially expressed genes (DEGs) (149), especially in different types of cancers, and leads to a varied immune microenvironment (**Figure 4**), although most scattered studies did not provide systematized and convincing results (**Table 3**).

**Table 3:** Biological functions of m^5^C-related genes in immune cells.

Immune cells and subgroups		Disease type	Associated m^5^C-related genes	Target genes	Biological functions	References
T cells	CD4+T cells	prostate cancer	NSUN2	/	/	(37)
		CRC	DNMT3A	/	/	(80)
		/	TET1, TET2	/	TET1 and TET2 convert m^5^C into its oxidative derivatives, regulating CTCF-dependent pre-mRNA splicing, which affects gene expression	(150)
		SLE	NSUN2	mRNA	NSUN2 levels decrease along with mRNA m^5^C levels of CD4+ T cells	(151)
		HIV-1 infection	NSUN1, NSUN2	/	NSUN1 suppresses viral replication; NSUN2 facilitates the methylation and replication of HIV-1 transcripts	(46, 152)
		/	NSUN2	IL-17A mRNA	NSUN2 enhances IL-17A secretion of T cells by methylating IL-17A mRNA at C466, stimulating its translation	(60)
	CD8+ T cells	/	NSUN3, NSUN6, TET1, TET3	/	/	(38, 66, 80)
B cells	memory B cells	prostate cancer	NSUN2	/	/	(37)
	naïve B cells	prostate cancer	NSUN6, TET1, TET3	/	/	(38)
	B cells	CRC	NSUN6, DNMT3A	/	/	(80)
		/	NSUN6	/	NSUN6 is dispensable for germinal center B-cell formation but necessary for the formation of antibody-secreting plasma cells	(83)
macrophages		prostate cancer	NSUN6, TET1, TET3	/	/	(38)
		HNSCC	NSUN3	/	NSUN3 promotes infiltration of M2 macrophages but suppresses infiltration of M1 macrophages	(64)
		AAA	ALYREF	lncRNAs	ALYREF-interacting lncRNAs are involved in immune system regulation and macrophage infiltration	(99)
neutrophils		lung squamous cell carcinoma	NSUN4	/	/	(66)
		/	TET2, TET3	socs3b mRNA	TET2 and TET3 influences neutrophil granulation, phagocytosis and cytokine signaling by demethylating and destabilizing socs3b mRNA	(153)
NK cells		prostate cancer	NSUN2	/	/	(37)
DC		CRC	DNMT3A	/	/	(80)

CTCF, CCCTC‐binding factor; socs3b, suppressors of cytokine signaling 3b.

### T cells

Subgroups of T lymphocytes (mainly CD4+ and CD8+ T cells) are distinguished in T cells by surface markers using flow cytometry (154). Upon leaving the thymus, naïve CD4+ T cells further differentiate into T cell subsets according to different stimulating signals, such as T helper (Th) cells (e.g., Th1, Th2, Th9, Th17, Th22), T follicular helper (Tfh) cells and regulatory T cells (Tregs) (155).

Recent studies have mainly focused on the m^5^C RNA modification of T cells in the context of cancer. For instance, the abundance of CD4+ T cells is higher in patients with soft tissue sarcoma (STS) (149), ccRCC (110) and HCC (148) but the opposite is found in glioma patients (156). Tregs appear to be slightly different from common CD4+ T cells, as they correlate with poor prognosis in STS (149) and HCC (138) patients and positive outcomes in HNSCC (147) and bladder cancer (140) patients. The role of CD8+ T cells in cancer is also controversial since they are considered protective factors in bladder cancer (140) and lung adenocarcinoma (157) patients but risk factors in ccRCC (110) patients.

For CD4+ T cells, associated m^5^C-related regulators include NSUN1 (46), NSUN2 (37, 60, 151, 152), DNMT3A (80), TET1 (150) and TET2 (150). Downregulated NSUN2 expression, along with decreased mRNA m^5^C levels of CD4+ T cells, was observed in systemic lupus erythematosus (SLE) patients, while the number of m^5^C-containing RNAs increased. In addition, m^5^C sites were mainly distributed in mRNA translation initiation sites, and hypermethylated m^5^C and/or upregulated genes in SLE were enriched immune-related and inflammatory pathways, including immune system signaling pathway, cytokine signaling pathway, and interferon signaling pathway (151). In HIV-1-infected CD4+ T cells, NSUN2, as the primary HIV-1 m^5^C methyltransferase, facilitates HIV-1 transcript methylation as well as viral replication (152). Note that NSUN2 inactivation did not reduce HIV-1 mRNA expression levels but did downregulate protein expression, suggesting the role of m^5^C in HIV-1 translation. Additionally, m^5^C loss dysregulates the alternative splicing of viral RNAs (152). In contrast, NSUN1 deficiency caused latently infected HIV-1 proviruses to reactivate, revealing the viral suppressive effects of NSUN1 (46). Moreover, NSUN2 enhances IL-17A secretion by T cells by methylating IL-17A mRNA at C466, stimulating its translation (60). Finally, the m^5^C erasers TET1 and TET2 regulate pre-mRNA splicing in a CCCTC‐binding factor (CTCF)-dependent manner.

Moreover, m^5^C-related regulators affecting CD8+ T cells include NSUN3, NSUN6, TET1 and TET3 (38, 66, 80), but no further studies were found.

### B cells and plasma cells

B cells are derived from hematopoietic stem cells (HSCs) in the bone marrow. Naïve B cells, once properly activated, mature into plasma cells, the antibody-secreting form of B cells, following an intrinsic developmental process (158). Recent studies on B cells mainly concentrated on alterations in m^5^C RNA modifications during tumor pathology. In HNSCC patients, a higher number of naïve B cells is negatively correlated with the risk score for poor prognosis (147). Activated plasma cells exert similar effects in bladder cancer (140) and lung adenocarcinoma (157) patients. Also, a prognostic model for pancreatic cancer based on m^6^A/m^5^C/m^1^A-associated lncRNAs showed that the low-risk group has a significantly higher concentration of naïve B cells and plasma cells within the TME (159), suggesting the protective role of B cells and plasma cells. Genes associated with m^5^C RNA modifications in B cells include NSUN2, NSUN6, DNMT3A, TET1 and TET3 (37, 38, 80). Specifically, although NSUN6 is dispensable for germinal center (GC) B-cell formation, it plays vital roles in the formation of antibody-secreting plasma cells (83).

### Macrophages

Monocytes and macrophages stem from hematological precursors in the bone marrow and are important in the innate immune system due to their phagocytic and antigen-presenting activity (160). Monocytes accumulate in peripheral blood, while macrophages are tissue-resident mature monocytes (161). Classically activated macrophages, or M1 macrophages, are proinflammatory, while alternatively activated macrophages, or M2 macrophages, are anti-inflammatory (162). Similar to T cells and B cells, most studies on m^5^C RNA modifications in macrophages are in the context of cancer development, especially macrophage infiltration in the TME. The risk scores based on m^5^C-related genes of patients with four types of cancer (HCC, HNSCC, glioma and pancreatic cancer) were positively correlated with infiltration of resting macrophages (M0), M1 or M2 macrophages (138, 147, 148, 156, 159). Analysis of the TME in prostate cancer patients showed differentially expressed NSUN6, TET1 and TET3 in M1 and M2 macrophages (38). NSUN3 promotes infiltration of M2 macrophages but suppresses M1 macrophage infiltration in HNSCC patients (64). Moreover, in AAA patients, ALYREF-interacting lncRNAs are involved in immune system processes and macrophage infiltration (99).

### Granulocytes

Granulocytes refer to a group of leukocytes with specific cytoplasmic granules distinguished by Romanowsky staining into three main subsets, namely, neutrophils, eosinophils and basophils (163). Currently, the scarcity of research focusing on m^5^C RNA modification of eosinophils and basophils makes it difficult to conclude m^5^C-related alterations in these two types of granulocytes. Only in lung adenocarcinoma patients is eosinophil infiltration correlated with a favorable prognostic pattern (157). Neutrophil abundance is generally associated with poor prognosis (138, 147), and a study on lung squamous cell carcinoma patients discovered that NSUN4 exerts a regulatory effect on neutrophil m^5^C RNA modification (66). Studies have also reported that TET2 and TET3 influence neutrophil granulation, phagocytosis and cytokine signaling by demethylating and destabilizing socs3b (suppressors of cytokine signaling 3b) mRNA, a member of the suppression of cytokine signaling gene family (153). TET2/3-defective embryos showed aberrant granule formation, defective phagocytosis and dysregulation of cytokine signaling in neutrophils due to accumulation of socs3b mRNA, which binds the Jak receptor to prevent Stat phosphorylation and downstream signaling *via* the Jak/Stat pathway (153).

### Others

Natural killer (NK) cells constitute a first line of innate immunity against tumors due to their capabilities of killing aberrant cells (164). Regarding m^5^C RNA modifications in NK cells, both resting and activated NK cells are correlated with positive outcomes in HNSCC (147), glioma (156) and lung adenocarcinoma (157) patients, with NSUN2 being the most closely associated m^5^C gene (37).

Dendritic cells (DCs) are generally regarded as the most potent antigen-presenting cells, thus modulating both immunity and tolerance (165). In lung adenocarcinoma patients, more DC infiltration was discovered in the low-risk group, suggesting the protective role of m^5^C in DCs (157). In contrast, a prognostic model for pancreatic cancer based on m^6^A/m^5^C/m^1^A-associated lncRNAs showed that the high-risk group has a significantly higher concentration of activated DCs within the TME (159), showing the tumor-promoting effects of activated DCs. The conflicting results might result from the differences in cancer types, DC subtypes, activation extent and patient characteristics. Consequently, rigorous future studies are needed in this regard. In CRC patients, the m^5^C writer DNMT3A was found to be involved in regulating DCs (80).

Mast cells are tissue-resident cells that function in inflammatory responses and tissue homeostasis (166). These cells are usually recognized clinically for their roles in IgE-mediated degranulation and allergic inflammation (167). A risk model based on m^6^A/m^1^A/m^5^C-regulated genes in HCC patients indicated the links between poor prognosis and high infiltration of resting mast cells (148).

In summary, the roles of m^5^C RNA modification of immune cells in the context of tumorigenesis largely remain to be further clarified. A better understanding of the mechanisms by which alterations in cell expression, infiltration and activation are regulated by methylation can be extremely helpful for the development of novel methods for tumor diagnosis as well as treatment.

## Summary and perspectives

In this review, we provide a detailed review concerning the roles of m^5^C RNA modifications in cancer by discussing m^5^C RNA-related genes and alterations in gene expression and immune cell infiltration. The modifications involve mainly mRNAs but also other noncoding RNAs such as tRNA, rRNA, and lncRNA, and are regulated by RNA m^5^C writers, readers and erasers, leading to changes in RNA processes, including transcription, transportation, translation and metabolism. Risk models made for prognosis prediction are based on m^5^C regulators as well as genes with m^5^C modification, which indicates the relationship between prognosis and alterations in immune cell infiltration in the TME.

However, numerous questions regarding oncogenic m^5^C RNA modifications remain to be elucidated. For example, the mechanisms by which m^5^C writers, readers and erasers function have only been investigated in a limited manner. What are the target genes of RNA m^5^C modifications? How are these genes linked to cell signal transduction and tumor malignancy? Additionally, we now know that RNA m^5^C modifications are related to immune cell infiltration within the TME, but scarce and controversial study results offer no comprehensive and fully convincing conclusions. What alterations do RNA m^5^C modifications cause indifferent groups of immune cells? How are these alterations linked to the progression of cancer? Is immune cell infiltration protective or destructive for patients with malignant tumors? These questions might provide deeper insights into the diagnosis and treatment of different cancer types.

Nevertheless, studies on RNA m^5^C modifications in cancer patients have massively progressed within the last five years, providing new analytical results from clinical samples. It should be pointed out that most of the studies based on clinical samples were only limited at the laboratory level, and no m^5^C-related clinical trials against cancer has been performed so far. However, as detection methods for RNA m^5^C sites continue to improve, cancerous RNA m^5^C modifications will most likely remain a popular scientific topic in the years to come and, hopefully, instill new hope for millions of patients fighting cancer.

## Author contributions

XG and XM have equal contributions to this study. XG and HZ designed the whole study. XG and XM drafted the manuscript. CC, JG, JW, and SW made the relevant edits to the manuscript. XG and XM revised the manuscript. All authors contributed to the article and approved the submitted version.
